# The metastasis patterns and their prognostic features in patients with de novo metastatic breast cancer of different ages

**DOI:** 10.1002/cam4.6509

**Published:** 2023-09-08

**Authors:** Shujuan Sun, Xiaochu Man, Dongdong Zhou, Fangchao Zheng, Jiuda Zhao, Xuesong Chen, Tong Liu, Jie Huang, Qiaorui Tan, Na Li, Huihui Li

**Affiliations:** ^1^ Department of Breast Medical Oncology Shandong Cancer Hospital and Institute, Shandong First Medical University and Shandong Academy of Medical Sciences Jinan China; ^2^ Breast Disease Diagnosis and Treatment Center of Affiliated Hospital of Qinghai University & Affiliated Cancer Hospital of Qinghai University Xining China; ^3^ Department of Breast Medical Oncology Harbin Medical University Cancer Hospital Harbin China; ^4^ Department of Breast Surgery Harbin Medical University Cancer Hospital Harbin China

**Keywords:** age, De novo metastatic breast cancer, metastasis pattern, prognosis

## Abstract

**Purpose:**

The prognostic outcomes of metastasis patterns in patients with de novo metastatic breast cancer (dnMBC) of different ages are unknown. Our study used a large‐scale data to investigate the metastasis patterns and prognostic features in dnMBC of different ages.

**Methods:**

Total 24,698 women with dnMBC in the Surveillance, Epidemiology and End Results database (2010–2018) were divided into three groups by age. Chi‐squared test was used to compare metastasis patterns and logistic regression was performed to investigate the risk of age and specific organ metastases. Kaplan–Meier survival curves were used to compare the overall survival.

**Results:**

In three groups, young group had the largest proportion of liver metastases (35.2% vs. 28.2% vs. 21.1%, *p* < 0.001), and elderly group had the largest proportion of lung metastases (22.6% vs. 30.0% vs. 35.0%, *p* < 0.001) and the lowest proportion of bone metastases (65.7% vs. 67.6% vs. 64.4%, *p* < 0.001). In young group, patients with liver metastases had better prognosis than patients with lung metastases (MST: 34 months vs. 29 months, *p* = 0.041), but in middle‐aged and elderly groups, the prognosis of lung metastases was better than that of liver metastases (MST in middle‐aged group: 24 months vs. 20 months, *p* = 0.002; MST in elderly group: 12 months vs. 6 months, *p* < 0.001).

**Conclusion:**

DnMBC patients at different age have distinct metastasis patterns and prognostic features. The findings lend support to consideration of tailored management and surveillance strategies for different age patients.

## 1 INTRODUCTION

Female breast cancer has become the most incident malignancy globally since 2020, and the leading cause of female cancer‐related death.^1^ As a consequence of development in early diagnosis and comprehensive therapeutic strategies, the prognosis of breast cancer has been improved markedly. However, distant metastasis remains a critical issue in breast cancer, with a five‐year relative survival rate of 28%,^2^ contributing to the majority of breast cancer‐related deaths. Breast cancer can metastasize to a variety of vital organs (such as bone, lung, liver, brain, etc.), exhibiting seemingly messy metastases, leading to differences in treatment response and prognoses of breast cancer patients.^3^, ^4^ Hence, knowledge of the metastasis patterns may promote to develop appropriate management and surveillance strategies for breast cancer.

Previous studies have shown significant differences in the risk of developing specific metastases in breast cancer at different ages. In a retrospective study of 9143 de novo metastatic breast cancer (dnMBC) patients, Leone et al. found that compared with patients <50 years old, patients >64 years old had a higher risk of developing lung metastases and a lower risk of developing bone and liver metastases.^5^ In addition, Xiao et al. showed that patients aged 40–65 years was related to higher risk of lung and brain metastases relative to <40 years.^6^ The consistent conclusions have also been found in other studies,^7^, ^8^, ^9^, ^10^ suggesting that breast cancer patients of different ages may have distinct metastasis patterns. However, the reports of metastasis patterns in patients with dnMBC of different ages are currently lacking.

Using the up‐to‐date data released from the SEER database, this study identified a large‐scale cohort of dnMBC between 2010 and 2018. According to the current guidelines,^11^, ^12^ individuals who were diagnosed with the condition were categorized into three age‐based groups: the young group (under 40 years), the middle‐aged group (between 40 and 65 years), and the elderly group (over 65 years). The aim was to analyze and compare the metastasis patterns and prognostic characteristics across different age groups.

## 2 PATIENTS AND METHODS

### 2.1 Study population

**FIGURE 1:**
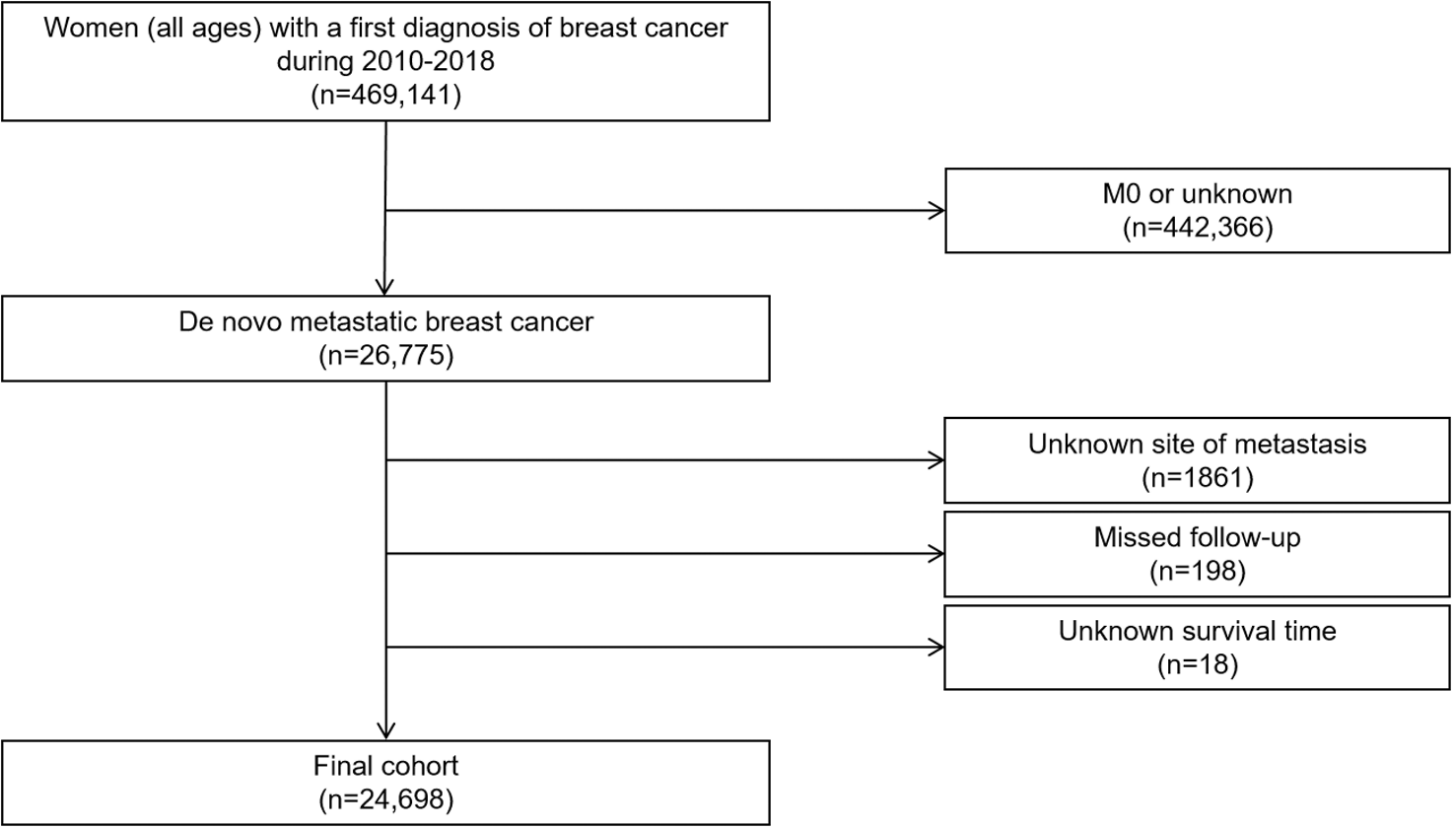
Flow diagram of patient selection.

Utilizing data from the SEER database, this study identified a cohort of female patients diagnosed with their first primary breast cancer. Given that information about metastatic site was not available in the SEER database until 2010, we selected this year as the study beginning point. Then, patients who were in M0 or unknown stage were excluded. And patients with unknown bone/brain/liver/lung metastatic status, unknown survival status, and unknown survival time were also excluded. Finally, a total of 24,698 dnMBC patients met the inclusion criteria and were included for final analysis. Detailed eligibility criteria was showed in Figure 1.

### 2.2 Data source

The SEER database provides an open access for tumor patients' demographic and clinical data as well as survival information. We selected the Incidence‐SEER Research Plus Data, which contained the 18 Registries (2000–2018), and released on April 15, 2021, based on the November 2020 submission, covering approximately 28% of the total population of the United States. We use the SEER*Stat software version 8.3.9 (http://www.seer.cancer.gov/seerstat) from the National Cancer Institute to download data. This database‐based retrospective study is exempt from the informed consent and approval of the institutional ethics committee. For this study, we signed the SEER research data‐use agreement to access the SEER 2000–2018 Research Plus Data file with the username 12,313‐Nov2020. Data was obtained following approved guidelines.

### 2.3 Variable definitions

Patient's information such as patient ID, age at diagnosis, race, grade, T stage, N stage, subtype, metastatic sites, survival, and follow‐up information were collected. The variables were classified as follows: race (white, black, others, and unknown), grade (I‐II, III‐IV, and unknown), T stage (T0‐1, T2, T3, T4, and Tx), and N stage (N0, N1, N2, N3, and Nx). According to the information provided by the SEER database, the distant metastatic organs in this study were limited to bone, lung, liver, and brain. In this study, the overall survival (OS) was used as the primary outcome measure.

### 2.4 Statistical analysis

Chi‐squared test was used to compare frequencies for categorical variables among groups. Multivariate logistic regression analyzes were performed to identify the risk of age and specific organ metastases, and the adjusted odds ratios (ORs) with 95% confidence interval (95% CI) were calculated. Kaplan–Meier survival curves were drawn with GraphPad Prism 9.3.1 and the curves were compared with log‐rank test. The 1‐year survival rate, 3‐year survival rate, and median survival time (MST) were obtained from survival tables. All the statistical analyses were carried out with the SPSS statistical software, version 22.0 (IBM Corp.). *p*‐Values below 0.05 were considered statistically significant.

## 3 RESULTS

### 3.1 Metastasis patterns in patients with dnMBC of different ages

Overall, of 469,141 women diagnosed with first primary breast cancer between 2010 and 2018, 26,775 were diagnosed with dnMBC, and among them 24,698 were finally included for analysis (Data S1). The eligible patients were classified into three groups based on age at diagnosis, among which 1776 patients (7.2%) were young group (<40 years), 13,254 patients (53.7%) were middle‐aged group (40–65 years), and 9668 patients (39.1%) were elderly group (>65 years).

As seen in Table 1, there was a clear contrast in the metastasis patterns among three age groups. Young group (<40 years) had the largest proportion of liver metastases than the middle‐aged and elderly (35.2% vs. 28.2% vs. 21.1%, *p* < 0.001). While elderly group (>65 years) had the largest proportion of lung metastases than the young and middle‐aged (22.6% vs. 30.0% vs. 35.0%, *p* < 0.001) and the lowest proportion of bone metastases than the young and middle‐aged (65.7% vs. 67.6% vs. 64.4%, *p* < 0.001). Surprisingly, the middle‐aged patients had the highest rate of brain metastases (6.5% vs. 8.2% vs. 6.0%, *p* < 0.001). We further compared the patterns of specific number and specific combination of metastases among three groups. It was found that the middle‐aged group had the lowest rate of one metastatic site (58.4% vs. 54.1% vs. 58.4%, *p* < 0.001) and the highest rate of multiple metastatic sites (two sites: 22.9% vs. 24.2% vs. 23.2%, *p* = 0.012; three sites: 6.2% vs. 8.5% vs. 6.1%, *p* < 0.001) than the other two groups. The common metastases in three age groups were only bone metastasis, only lung metastasis, only liver metastasis, both bone and lung metastases, and both bone and liver metastases，all of which accounted for more than 70% of all age groups. Except the only bone metastasis, the incidence of other four metastases combinations were significant different among three age groups (*p* < 0.001).

**TABLE 1:** Metastasis patterns in patients with de novo metastatic breast cancer of different ages.

Metastatic sites	Age at diagnosis			Total *n* = 24, 698 (%)	*p‐*Value
	<40 years *n* = 1776 (7.2%)	40–65 years *n* = 13,254 (53.7%)	>65 years *n* = 9668 (39.1%)		
Bone metastases	1167 (65.7)	8957 (67.6)	6224 (64.4)	16,348 (66.2)	<0.001
Lung metastases	401 (22.6)	3971 (30.0)	3380 (35.0)	7752 (31.4)	<0.001
Liver metastases	626 (35.2)	3741 (28.2)	2041 (21.1)	6408 (25.9)	<0.001
Brain metastases	116 (6.5)	1084 (8.2)	582 (6.0)	1782 (7.2)	<0.001
One site	1038 (58.4)	7166 (54.1)	5650 (58.4)	13,854 (56.1)	<0.001
Bone‐only	665 (37.4)	4925 (37.2)	3681 (38.1)	9271 (37.5)	0.367
Lung‐only	134 (7.5)	1100 (8.3)	1300 (13.4)	2534 (10.3)	<0.001
Liver‐only	218 (12.3)	952 (7.2)	550 (5.7)	1720 (7.0)	<0.001
Brain‐only	21 (1.2)	189 (1.4)	119 (1.2)	329 (1.3)	0.378
Two sites	407 (22.9)	3214 (24.2)	2241 (23.2)	5862 (23.7)	0.012
Bone+lung	104 (5.9)	1241 (9.4)	1116 (11.5)	2461 (10.0)	<0.001
Bone+liver	235 (13.2)	1271 (9.6)	629 (6.5)	2135 (8.6)	<0.001
Lung+liver	34 (1.9)	323 (2.4)	267 (2.8)	624 (2.5)	0.070
Bone+brain	24 (1.4)	246 (1.9)	145 (1.5)	415 (1.7)	0.062
Lung+brain	6 (0.3)	98 (0.7)	67 (0.7)	171 (0.7)	0.159
Liver+brain	4 (0.2)	35 (0.3)	17 (0.2)	56 (0.2)	0.382
Three sites	110 (6.2)	1129 (8.5)	593 (6.1)	1832 (7.4)	<0.001
Bone+lung+liver	81 (4.6)	806 (6.1)	438 (4.5)	1325 (5.4)	<0.001
Bone+lung+brain	7 (0.4)	162 (1.2)	94 (1.0)	263 (1.1)	0.003
Bone+liver+brain	19 (1.1)	113 (0.9)	42 (0.4)	174 (0.7)	<0.001
Lung+liver+brain	3 (0.2)	48 (0.4)	19 (0.2)	70 (0.3)	0.043
Four sites	32 (2.0)	193 (1.5)	79 (0.8)	304 (1.2)	<0.001
Other sites	189 (10.6)	1552 (11.7)	1105 (11.4)	2846 (11.5)	0.389

*Note*: *p*‐Values represent the comparison between patients <40 years, 40–65 years, and >65 year.

### 3.2 Association between age and organ‐specific metastases

Using univariate and multivariate logistic regression analyzes, we further confirmed these observations about the relationship between age and specific organ metastases (Table 2). Age <40 years old was used as reference, after adjusted for race, marital status, grade, T stage, N stage, and subtype, it was found that older age (>65 years) was associated with higher risk of lung metastases (OR: 1.980, 95% CI: 1.746–2.245), but lower risk of bone (OR: 0.790, 95% CI: 0.704–0.887) and liver (OR: 0.565, 95% CI: 0.502–0.635) metastases. As shown in Table 2, middle age (40–65 years) is independently associated with increased prevalence of lung metastases (OR: 1.511, 95% CI: 1.340–1.705) and brain metastases (OR: 1.281, 95% CI: 1.047–1.568), while associated with decreased prevalence of liver metastases (OR: 0.797, 95% CI: 0.715–0.888). In addition to this, we found some results regarding the relationship between subtypes and specific organ metastases. Using HR+/HER2− as reference, after adjusting for age, race, marital status, grade, T stage, and N stage, we found that HR+/HER2+, HR−/HER2+, and HR−/HER2− all increased the risk of lung, liver, and brain metastases; however, decreased the risk of bone metastases. Among the subtypes, HR+/HER2+ was associated with the lowest risk of bone metastases (OR: 0.320, 95% CI: 0.293–0.349); HR−/HER2+ was independently associated with the highest risk of liver (OR: 3.187, 95% CI: 2.876–3.531) and brain metastases (OR: 2.111, 95% CI: 1.787–2.493). We also performed univariate and multivariate logistic regression analyzes to determine the relationship between HR+ and age on metastatic patterns (Table 3). Age <40 years old was used as reference; middle‐aged (40–65 years) patients were independently associated with increased odds of lung (OR: 1.545, 95% CI: 1.323–1.805) and brain metastasis (OR: 1.470, 95% CI: 1.099–1.967) and decreased odds of liver metastasis (OR: 0.773, 95% CI: 0.676–0.884); older age (>65 years) patients would be independently associated with increased odds of lung metastasis (OR: 2.065, 95% CI: 1.757–2.426) and decreased odds of bone (OR: 0.506, 95% CI: 0.437–0.586) and liver metastasis (OR: 0.768, 95% CI: 0.663–0.890).

**TABLE 2:** Multivariate logistic regression for metastatic sites in de novo metastatic breast cancer.

Variable	Bone metastases		Lung metastases		Liver metastases		Brain metastases	
	Univariate OR (95% CI) *p*‐value	Multivariate OR (95% CI) *p*‐value	Univariate OR (95% CI) *p*‐value	Multivariate OR (95% CI) *p*‐value	Univariate OR (95% CI) *p*‐value	Multivariate OR (95% CI) *p*‐value	Univariate OR (95% CI) *p*‐value	Multivariate OR (95% CI) *p*‐value
Age								
<40 years	1 (reference)	1 (reference)	1 (reference)	1 (reference)	1 (reference)	1 (reference)	1 (reference)	1 (reference)
40–65 years	1.083 (0.976, 1.203) 0.13387	0.986 (0.883, 1.100) 0.79757	**1.456 (1.294, 1.637) < 0.00001**	**1.511 (1.340, 1.705) < 0.00001**	**0.736(0.662, 0.817) < 0.00001**	**0.797 (0.715, 0.888) 0.00004**	**1.257 (1.031, 1.534) 0.02395**	**1.281 (1.047, 1.568) 0.01596**
>65 years	0.956 (0.860, 1.063) 0.40535	**0.790 (0.704, 0.887) 0.00007**	**1.833 (1.628, 2.063) < 0.00001**	**1.980 (1.746, 2.245) < 0.00001**	**0.492 (0.441, 0.548) < 0.00001**	**0.565 (0.502, 0.635) < 0.00001**	0.959 (0.782, 1.177) 0.68979	0.999 (0.805, 1.238) 0.98990
Race								
White	1 (reference)	1 (reference)	1 (reference)	1 (reference)	1 (reference)	1 (reference)	1 (reference)	1 (reference)
Black	**0.774 (0.722, 0.830) < 0.00001**	**0.842 (0.782, 0.907) < 0.00001**	**1.306 (1.217, 1.402) < 0.00001**	**1.198 (1.112, 1.290) < 0.00001**	**1.159 (1.075, 1.250) 0.00012**	1.054 (0.974, 1.141) 0.19429	1.069 (0.941, 1.213) 0.30525	0.937 (0.822, 1.069) 0.33672
Others	**0.841 (0.765, 0.924) 0.00033**	**0.828 (0.750, 0.914) 0.00018**	**1.189 (1.080, 1.309) 0.00043**	**1.207 (1.094, 1.333) 0.00019**	**1.118 (1.009, 1.238) 0.03287**	1.021 (0.919, 1.135) 0.69469	0.927 (0.774, 1.111) 0.41339	0.899 (0.749, 1.080) 0.25461
Unknown	0.773 (0.535, 1.119) 0.17237	0.704 (0.480, 1.032) 0.07214	1.164 (0.795, 1.703) 0.43575	1.261 (0.855, 1.862) 0.24240	0.817 (0.529, 1.263) 0.36314	0.716 (0.457, 1.122) 0.14524	0.561 (0.229, 1.375) 0.20646	0.496 (0.201, 1.223) 0.12778
Grade								
I–II	1 (reference)	1 (reference)	1 (reference)	1 (reference)	1 (reference)	1 (reference)	1 (reference)	1 (reference)
III–IV	**0.456 (0.427, 0.487) < 0.00001**	**0.610 (0.568, 0.654) < 0.00001**	**1.442 (1.350, 1.540) < 0.00001**	**1.251 (1.165, 1.343) < 0.00001**	**1.607 (1.497, 1.724) < 0.00001**	**1.293 (1.199, 1.395) < 0.00001**	**1.472 (1.301, 1.666) < 0.00001**	**1.169 (1.026, 1.333) 0.01933**
Unknown	**0.731 (0.683, 0.782) < 0.00001**	**0.849 (0.788, 0.914) 0.00001**	**1.241 (1.160, 1.328) < 0.00001**	**1.092 (1.015, 1.175) 0.01876**	**1.315 (1.223, 1.413) < 0.00001**	**1.161 (1.073, 1.256) 0.00021**	**1.565 (1.383, 1.770) < 0.00001**	**1.317 (1.154, 1.503) 0.00004**
T stage								
T0‐1	1 (reference)	1 (reference)	1 (reference)	1 (reference)	1 (reference)	1 (reference)	1 (reference)	1 (reference)
T2	1.050 (0.957, 1.152) 0.30332	1.087 (0.986, 1.197) 0.09284	**1.310 (1.179, 1.455) < 0.00001**	**1.309 (1.177, 1.457) < 0.00001**	**1.134 (1.023, 1.256) 0.01631**	**1.120 (1.008, 1.246) 0.03555**	**0.771 (0.648, 0.917) 0.00334**	**0.788 (0.661, 0.940) 0.00826**
T3	0.997 (0.899, 1.105) 0.94749	**1.121 (1.005, 1.251) 0.04032**	**1.691 (1.509, 1.895) < 0.00001**	**1.670 (1.486, 1.877) < 0.00001**	1.117 (0.997, 1.253) 0.05720	1.073 (0.952, 1.209) 0.24730	0.864 (0.712, 1.047) 0.13657	0.856 (0.702, 1.043) 0.12232
T4	**0.912 (0.833, 0.999) 0.04768**	1.100 (0.998, 1.212) 0.05380	**2.556 (2.310, 2.827) < 0.00001**	**2.427 (2.186, 2.695) < 0.00001**	**1.151 (1.040, 1.273) 0.00644**	1.096 (0.985, 1.219) 0.09172	1.097 (0.932, 1.292) 0.26698	1.042 (0.879, 1.235) 0.63587
Tx	**1.184 (1.067, 1.314) 0.00147**	**1.255 (1.119, 1.406) 0.00010**	**1.593 (1.422, 1.785) < 0.00001**	**1.429 (1.266, 1.612) < 0.00001**	**1.326 (1.185, 1.483) < 0.00001**	**1.253 (1.110, 1.414) 0.00027**	**1.218 (1.017, 1.458) 0.03233**	1.079 (0.890, 1.309) 0.43754
N stage								
N0	1 (reference)	1 (reference)	1 (reference)	1 (reference)	1 (reference)	1 (reference)	1 (reference)	1 (reference)
N1	1.003 (0.936, 1.076) 0.92749	1.040 (0.966, 1.120) 0.30083	**1.302 (1.212, 1.399) < 0.00001**	**1.180 (1.095, 1.272) 0.00001**	**1.118 (1.038, 1.205) 0.00344**	1.007 (0.930, 1.089) 0.87145	1.037 (0.912, 1.179) 0.58275	0.991 (0.868, 1.132) 0.89429
N2	0.929 (0.842, 1.026) 0.14498	0.979 (0.880, 1.088) 0.68754	**1.202 (1.086, 1.331) 0.00038**	0.996 (0.895, 1.109) 0.94403	0.981 (0.880, 1.093) 0.72734	**0.873 (0.779, 0.979) 0.02018**	0.980 (0.814, 1.180) 0.83421	0.947 (0.781, 1.148) 0.57945
N3	**0.700 (0.641, 0.765) < 0.00001**	**0.795 (0.723, 0.874) < 0.00001**	1.054 (0.960, 1.158) 0.27061	**0.834 (0.754, 0.921) 0.00037**	**0.849 (0.768, 0.939) 0.00143**	**0.707 (0.635, 0.787) < 0.00001**	1.002 (0.847, 1.185) 0.98303	0.889 (0.746, 1.060) 0.18889
Nx	1.075 (0.972, 1.190) 0.15996	1.067 (0.955, 1.192) 0.25285	**1.246 (1.125, 1.380) 0.00002**	1.115 (0.998, 1.245) 0.05411	**1.192 (1.073, 1.325) 0.00112**	1.093 (0.974, 1.227) 0.12863	**1.361 (1.148, 1.615) 0.00040**	1.172 (0.975, 1.410) 0.09041
Subtype								
HR+/HER2−	1 (reference)	1 (reference)	1 (reference)	1 (reference)	1 (reference)	1 (reference)	1 (reference)	1 (reference)
HR+/HER2+	**0.269 (0.248, 0.292) < 0.00001**	**0.320 (0.293, 0.349) < 0.00001**	**1.752 (1.612, 1.903) < 0.00001**	**1.604 (1.468, 1.752) < 0.00001**	**1.615 (1.474, 1.771) < 0.00001**	**1.487 (1.351, 1.637) < 0.00001**	**2.124 (1.845, 2.444) < 0.00001**	**2.032 (1.753, 2.355) < 0.00001**
HR−/HER2+	**0.310 (0.282, 0.342) < 0.00001**	**0.346 (0.313, 0.383) < 0.00001**	**1.416 (1.281, 1.566) < 0.00001**	**1.334 (1.201, 1.481) < 0.00001**	**3.518 (3.185, 3.886) < 0.00001**	**3.187 (2.876, 3.531) < 0.00001**	**2.233 (1.900, 2.624) < 0.00001**	**2.111 (1.787, 2.493) < 0.00001**
HR−/HER2−	**0.644 (0.595, 0.697) < 0.00001**	**0.684 (0.631, 0.742) < 0.00001**	**1.155 (1.065, 1.252) 0.00046**	**1.137 (1.046, 1.236) 0.00249**	**2.350 (2.167, 2.548) < 0.00001**	**2.167 (1.995, 2.354) < 0.00001**	**1.544 (1.337, 1.782) < 0.00001**	**1.500 (1.297, 1.736) < 0.00001**
Unknown	**0.592 (0.546, 0.641) < 0.00001**	**0.584 (0.535, 0.637) < 0.00001**	**1.445 (1.334, 1.564) < 0.00001**	**1.367 (1.253, 1.492) < 0.00001**	**1.641 (1.506, 1.789) < 0.00001**	**1.562 (1.422, 1.715) < 0.00001**	**1.676 (1.454, 1.933) < 0.00001**	**1.417 (1.214, 1.653) < 0.00001**
Radiation								
No/unknown	1 (reference)	1 (reference)	1 (reference)	1 (reference)	1 (reference)	1 (reference)	1 (reference)	1 (reference)
Yes	**1.792 (1.688, 1.903) < 0.00001**	**1.853 (1.739, 1.974) < 0.00001**	**0.686 (0.646, 0.729) < 0.00001**	**0.722 (0.678, 0.768) < 0.00001**	**0.629 (0.589, 0.671) < 0.00001**	**0.603 (0.564, 0.645) < 0.00001**	**4.139 (3.745, 4.575) < 0.00001**	**4.649 (4.190, 5.159) < 0.00001**
Chemotherapy								
No/unknown	1 (reference)	1 (reference)	1 (reference)	1 (reference)	1 (reference)	1 (reference)	1 (reference)	1 (reference)
Yes	**0.746 (0.707, 0.787) < 0.00001**	**0.849 (0.798, 0.905) < 0.00001**	**0.915 (0.867, 0.966) 0.00131**	0.941 (0.883, 1.001) 0.05481	**1.428 (1.347, 1.514) < 0.00001**	**1.145 (1.070, 1.225) 0.00009**	0.968 (0.878, 1.066) 0.50568	**0.813 (0.728, 0.909) 0.00028**

*Note*: Only statistically significant results were presented (*p* < 0.05). Meaningful results have been highlighted in bold in the table.

**TABLE 3:** Multivariate logistic regression for metastatic sites in HR+ dnMBC.

Variable		Bone metastases		Lung metastases		Liver metastases		Brain metastases	
		Univariate OR (95% CI) *p*‐value	Multivariate OR (95% CI) *p*‐value	Univariate OR (95% CI) *p*‐value	Multivariate OR (95% CI) *p*‐value	Univariate OR (95% CI) *p*‐value	Multivariate OR (95% CI) *p*‐value	Univariate OR (95% CI) *p*‐value	Multivariate OR (95% CI) *p*‐value
HR+	<40 years	1 (reference)	1 (reference)	1 (reference)	1 (reference)	1 (reference)	1 (reference)	1 (reference)	1 (reference)
	40–65 years	1.049 (0.914, 1.205) 0.49317	0.987 (0.858, 1.136) 0.85796	**1.527 (1.311, 1.778) < 0.00001**	**1.545 (1.323, 1.805) < 0.00001**	**0.753 (0.659, 0.859) 0.00003**	**0.773 (0.676, 0.884) 0.00018**	**1.478 (1.107, 1.973) 0.00798**	**1.470 (1.099, 1.967) 0.00946**
	>65 years	0.878 (0.764, 1.009) 0.06749	**0.768 (0.663, 0.890) 0.00046**	**1.977 (1.696, 2.305) < 0.00001**	**2.065 (1.757, 2.426) < 0.00001**	**0.479 (0.417, 0.549) < 0.00001**	**0.506 (0.437, 0.586) < 0.00001**	1.192 (0.887, 1.602) 0.24380	1.236 (0.910, 1.680) 0.17443
HR−	<40 years	1 (reference)	1 (reference)	1 (reference)	1 (reference)	1 (reference)	1 (reference)	1 (reference)	1 (reference)
	40–65 years	0.992 (0.817, 1.204) 0.93248	0.978 (0.802, 1.191) 0.82223	**1.335 (1.083, 1.647) 0.00693**	**1.381 (1.113, 1.713) 0.00338**	**0.690 (0.567, 0.839) 0.00020**	**0.685 (0.560, 0.836) 0.00021**	1.249 (0.905, 1.724) 0.17559	1.164 (0.838, 1.615) 0.36544
	>65 years	0.869 (0.709, 1.065) 0.17694	0.859 (0.692, 1.066) 0.16689	**1.822 (1.466, 2.266) < 0.00001**	**1.854 (1.469, 2.339) < 0.00001**	**0.534 (0.434, 0.657) < 0.00001**	**0.500 (0.400, 0.624) < 0.00001**	0.930 (0.659, 1.312) 0.67952	0.789 (0.548, 1.135) 0.20184

*Note*: Only statistically significant results were presented (*p* < 0.05). Meaningful results have been highlighted in bold in the table.

We used univariate and multivariate logistic regression analyzes to determine the relationship between age and treatment on metastatic patterns (Table S1). For example, radiotherapy was independently associated with an increased risk of bone metastases, especially in older age (>65 years) patients (OR: 2.256, 95% CI: 2.028–2.510). Chemotherapy was independently associated with a reduced risk of bone metastases, especially in young age (<40 years) patients (OR: 0.601, 95% CI: 0.430–0.840). In addition, radiotherapy was independently associated with a reduced risk of lung metastases and liver metastases and an increased risk of brain metastases in patients of all ages. And chemotherapy was independently associated with an increased chance of liver metastases (OR: 1.203, 95% CI: 1.082–1.338) in older age (>65 years) patients and a decreased chance of brain metastases (OR: 0.761, 95% CI: 0.658–0.881) in the middle‐aged (40–65 years) patients. We also performed univariate and multivariate logistic regression analyzes to determine the relationship between subtype and treatment on the pattern of metastasis (Table S2). Similar to the results obtained in Table S1, radiotherapy was independently associated with an increased risk of bone and brain metastases and a decreased risk of lung and liver metastases. And there was little variation between subtypes. In particular, HR−/HER2+ was independently associated with a higher risk of brain metastasis (OR: 9.084, 95% CI: 6.461–12.772) compared to other subtypes. Under the influence of chemotherapy, HR+/HER2− were independently associated with a decreased risk of bone metastases (OR: 0.799, 95% CI: 0.733–0.872) and an increased risk of liver metastases (OR: 1.441, 95% CI: 1.309–1.586); HR−/HER2+ was independently associated with a decreased risk of lung metastases (OR: 0.66, 95% CI: 0.518–0.861) and liver metastases (OR: 0.622, 95% CI: 0.483–0.801); and HR−/HER2− and HR+/HER2+ were independently associated with a decreased risk of brain metastases (OR: 0.582, 95% CI: 0.440–0.771; OR: 0.620, 95% CI: 0.472–0.816).

### 3.3 Prognostic features of metastasis patterns in patients with dnMBC of different ages

**FIGURE 2:**
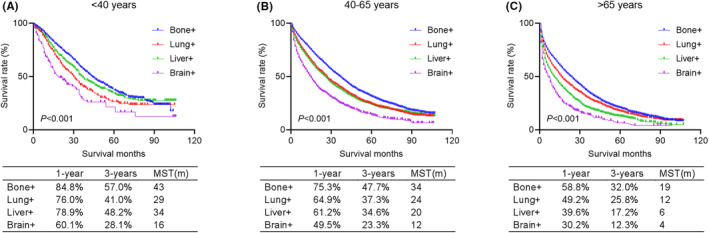
Kaplan–Meier curves comparing overall survival for specific organ metastases (bone+, lung+, liver+, and brain+) in young (A), middle‐aged (B), and elderly (C) groups, respectively.

We used Kaplan–Meier analysis and log‐rank test to investigate the prognostic features of specific organ metastases in dnMBC (Figure 2). Among all age groups, bone metastases had the best prognosis and brain metastases had the worst prognosis. While the prognostic features of lung metastases and liver metastases differed among three age groups. The results showed that young group patients with liver metastases (MST 34 months) had better prognosis than lung metastases (MST 29 months); (*p* = 0.041), while in middle‐aged and elderly groups, patients with lung metastases had better prognosis than liver metastases (MST in middle‐aged group: 24 months vs. 20 months, *p* = 0.002; MST in elderly group: 12 months vs. 6 months, *p* < 0.001).

**FIGURE 3:**
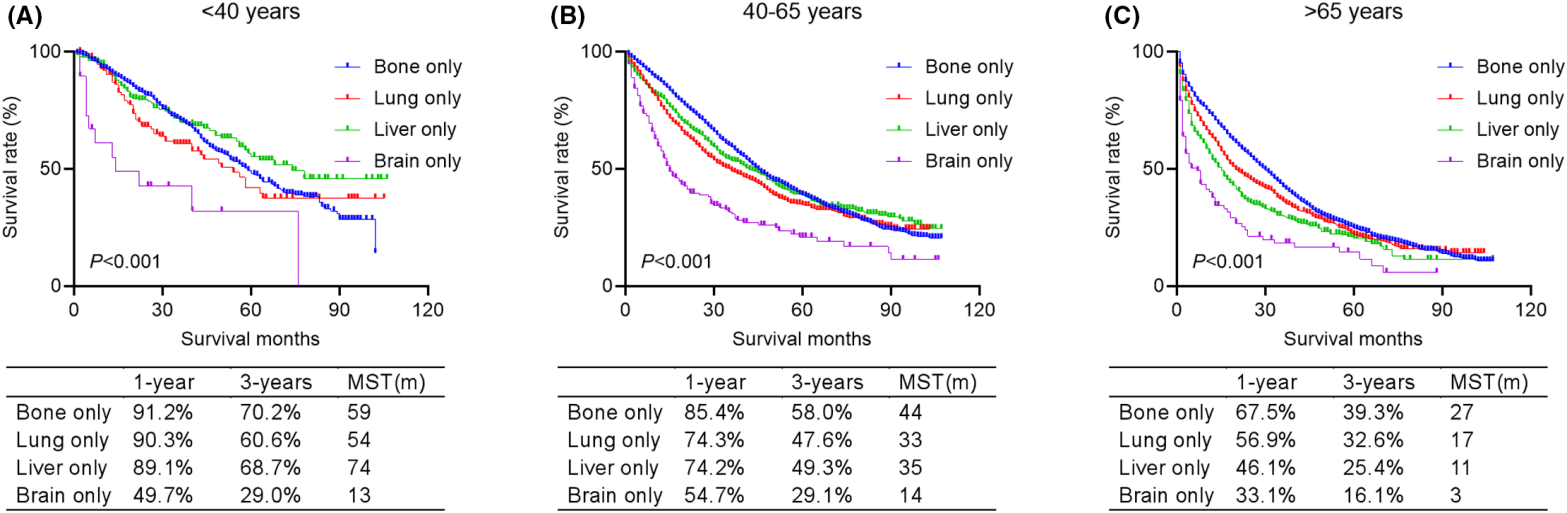
Kaplan–Meier curves comparing overall survival for simple metastatic site (bone‐only, lung‐only, liver‐only, and brain‐only) in young (A), middle‐aged (B), and elderly (C) groups, respectively.

We further investigated the prognostic features of simple metastatic site in dnMBC (Figure 3). The brain‐only metastasis still had the worst prognosis in all age groups. But the prognosis of bone‐only metastasis, lung‐only metastasis, and liver‐only metastasis in young group had no significant difference (*p* = 0.211). In middle‐aged and elderly groups, patients with bone‐only metastasis had the best prognosis. The prognosis of lung‐only metastasis and liver‐only metastasis were not significantly different in middle‐aged group (MST in middle‐aged group: 33 months vs. 35 months, *p* = 0.838), while in elderly group lung‐only metastasis had a significantly better prognosis than liver‐only metastasis (MST in elderly group: 17 months vs. 11 months, *p* < 0.001).

**FIGURE 4:**
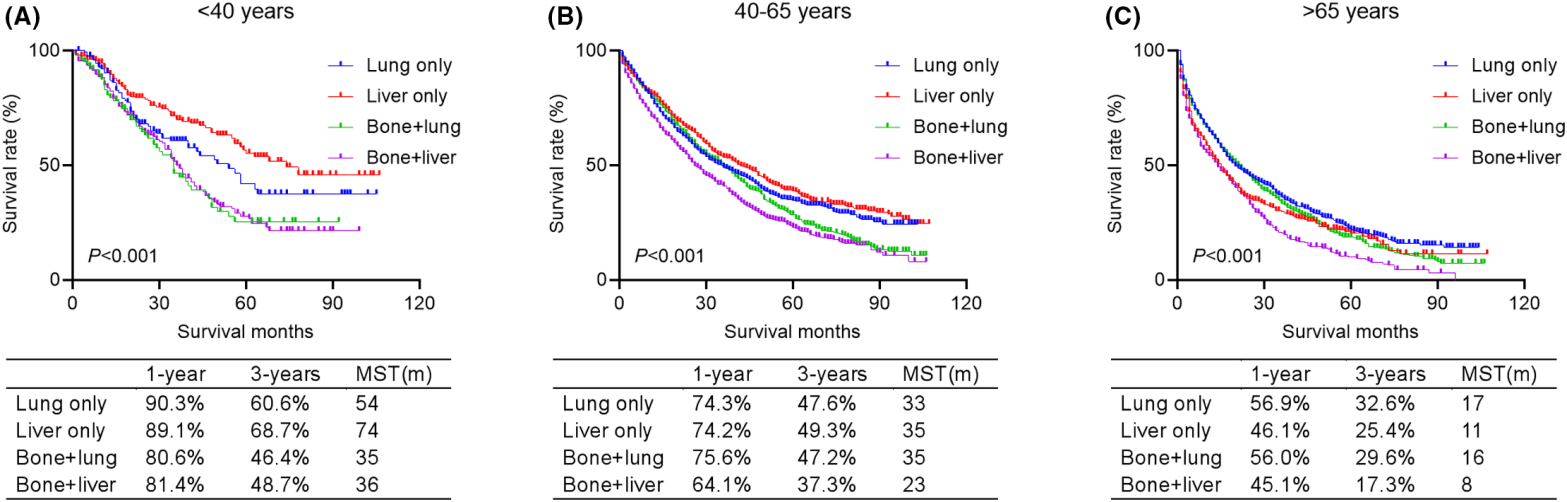
Kaplan–Meier curves comparing overall survival for specified metastases (lung‐only, liver‐only, bone+lung, and bone+liver) in young (A), middle‐aged (B), and elderly (C) groups, respectively.

In addition, we compared the prognosis of lung‐only metastasis, liver‐only metastasis, bone+lung metastases, bone+liver metastases in each age group (Figure 4). In the young and middle‐aged groups, lung‐only metastasis and liver‐only metastasis had better prognosis than bone+lung metastases and bone+liver metastases. And there was no significant difference in the prognosis of bone+lung metastases and bone+liver metastases in the young group, while the prognosis of bone+lung metastases in the middle‐aged group was better than that of bone+liver metastases. In the elderly group; however, lung‐only metastasis and bone+lung metastases had best prognoses, followed by liver‐only metastasis and bone+liver metastases.

Finally, we used Kaplan–Meier analysis to investigate the prognostic characteristics of different age groups in hormone receptor positivity patients (Figure S1). The results showed the best prognosis for young age (<40 years) patients and the worst prognosis for older age (>65 years) patients. We used Kaplan–Meier analysis to examine the prognostic characteristics of different treatments among patients (Figure S2). The results showed the best prognosis for the combination of radiotherapy and chemotherapy, with the same median survival for radiotherapy only and chemotherapy only (MST 20 months).

## 4 DISCUSSION

It is estimated that 5% of patients were diagnosed with metastatic breast cancer at the initial diagnosis, and with the 5‐year relative survival was only 26%.^13^ Knowledge of metastasis patterns is of great value to treatment and management for breast cancer. This study identified a large cohort of 24,698 female dnMBC patients from 2010 to 2018 to investigate the metastasis patterns and their prognostic features in patients with dnMBC by age groups.

Prior works have demonstrated that metastatic breast cancer is a complicated heterogeneous disease, comprising a series of biological characteristics, genomic alterations, metastatic behaviors, and prognostic outcomes.^14^, ^15^ This study found significant differences in dnMBC metastasis patterns at different ages, especially in lung involved metastases and liver involved metastases. And the logistic analyzes proved that compared with patients <40 years old, patients at 40–65 years old, or >65 years old have significantly higher risk of developing lung metastases and lower risk of developing liver metastases. Similar findings were also shown in previous studies.^9^, ^16^ But there were some inconsistent results regarding the risk of bone metastases and brain metastases. This result showed patients >65 years old have an increased prevalence of bone metastases and patients at 40–65 years old have a decreased prevalence of brain metastases, compared with patients <40 years old. Xiao et al. investigated the overall dnMBC population from 2010 to 2014 in the SEER database and demonstrated no significant association of older age (>65 years) with bone metastases,^6^ and another study using a cohort of dnMBC from 2010 to 2013 in the SEER database showed no significant correlation between middle age (50–64 years) and brain metastases.^5^ The possible reason for the controversial findings may be the limited number of patients in previous studies.

Besides the clinical research, a lot of basic and translational research were focused on the mechanisms of age and organ‐specific metastases. The mouse model of melanoma further supported the observation that older age was associated with higher rate of lung metastases, it was found that aged microenvironment can promote tumor lung metastasis in the mouse model of melanoma, which is related to the significant increase in sFRP2 secreted by aged fibroblasts.^17^ In addition, a transcriptomics analysis showed that the transcriptome changes in age and breast cancer‐related genes (DYNLT3, P4HA3, and LX4) during aging may contribute to the progression of breast cancer.^18^ Chatsirisupachai et al. performed a multi‐omics analysis to investigate age‐related genomic differences across cancer types, found that tumors from older patients present an overall increase in genomic instability, somatic copy number alterations, and somatic mutations.^19^ But there is still no findings about the genetic variations related to the organ‐specific metastases. Aging is a complicated process, accompanied by the aging of organ microenvironment and the accumulation of genetic mutations. The results provide an association between age and organ‐specific metastases, but the underlying mechanisms and gene expression remain largely unclear and require further exploration.

There are many studies focusing on the effect of age on the prognosis of breast cancer patients,^20^, ^21^, ^22^ but as far as we known, no study has investigated the prognostic features of metastasis patterns in patients of different ages, which the results showed they were distinct from each other. Mainly in case of liver metastases and lung metastases, patients with liver metastases had better prognosis than patients with lung metastases in the young group, but in the middle‐aged and elderly groups, the prognosis of patients with lung metastases was better than that of patients with liver metastases. The results showed that metastasis patterns at different ages have different prognostic outcomes, suggesting that more attention should be paid to the exploration of precise treatment in breast cancer patients.

Consistent with some previous studies, we found that the HR+/HER2− subtype had a tendency to cause bone metastases compared to other subtypes.^23^, ^24^, ^25^ And some findings suggest that COX‐2 inhibitors may reduce the risk of bone metastases in stage II and III breast cancer.^26^ In addition to this, several studies have shown the importance of bisphosphonates in the treatment of bone metastases.^27^ What's more, lung‐liver‐brain metastasis sites are also very common. In this study, HER2‐positive and HR−/HER2− subtypes were more likely to have lung metastases, liver metastases, and brain metastases compared to HR+/HER2− subtype. There are many findings^6^, ^28^, ^29^, ^30^, ^31^ that are consistent with the observations made in our study. Xiao W et al.^6^ showed that both HER2+ subtypes were associated with significantly higher liver, brain, and lung metastasis rates compared to HR+/HER2− tumors, but with progressively lower ORs in liver, brain, and lung metastasis rates. Triple‐negative tumors had a higher incidence of brain metastases (OR, 1.95), liver metastases (OR, 1.35), and lung metastases (OR, 1.34), but significantly lower bone metastases (OR, 0.64) compared to HR+/HER2− tumors. Recent studies have shown that the signal of focal adhesion is decreased in tumors of patients with lung recurrence,^30^, ^32^, ^33^ while up to 75.8% of all lung cancer patients with first distant metastases have EGFR‐positive or HER2‐positive. EGFR helps to promote the migratory and aggressive activity of tumor cells, while HER2 plays a key role in promoting internal fluid leakage from tumor cells.^34^ In addition, Li YM et al. found that CXCR4 was involved in promoting the invasion of cancer cells to internal organs, while HER2 enhanced the expression of CXCR4.^35^ Similarly, the WNT pathway was associated with brain recurrence in patients. The results suggest that active WNT/β‐catenin protein signaling contributes to the metastasis of basal breast tumors to the brain.^30^ So the differential patterns of metastasis and survival in different breast cancer subtypes are likely due to different gene and protein expression profiles.

Some limitations in this study need to be considered when interpreting the data presented. First, this is a retrospective observational study with some potential selection bias and confounding bias. Second, the information for sites of distant metastases was not comprehensive, some metastatic organs (such as pleura, peritoneum, etc.) were not documented in the SEER database. Besides, the information about metastasis loci is unclear in the SEER database, so we are unable to analyze any metastasis burden or the number of metastases information, which may offer greater value for understanding biological behavior. In addition, aging is a complicated and heterogeneous process,^36^ and factors like comorbidity, functional status, cognition, depression, nutrition, etc. play an important role in geriatric assessment of older patients.^23^ There was no exact definition for the age range of older age, and this study set the range of older age to >65 years, all interpretations in this study should build on this basis. The findings may provide some references for different age patients to develop tailored screening and treatment strategies.

## 5 CONCLUSION

In summary, this large‐scale population‐based study provided a reliable description of dnMBC metastasis patterns and their prognostic characteristics at different ages and showed an age‐specific risk of organ metastases in breast cancer patients.

## AUTHOR CONTRIBUTIONS

**Shujuan Sun:** Data curation (equal); formal analysis (equal); writing – original draft (equal); writing – review and editing (equal). **Xiaochu Man:** Writing – review and editing (equal). **Dongdong Zhou:** Writing – review and editing (equal). **Fangchao Zheng:** Writing – review and editing (equal). **Jiuda zhao:** Writing – review and editing (equal). **Xuesong Chen:** Writing – review and editing (equal). **Tong Liu:** Writing – review and editing (equal). **Jie Huang:** Writing – review and editing (equal). **Qiaorui Tan:** Writing – review and editing (equal). **Na Li:** Writing – review and editing (equal). **Huihui Li:** Conceptualization (equal); project administration (equal); supervision (equal).

## FUNDING INFORMATION

This work was supported by Shandong Provincial Natural Science Foundation Anticancer and Prevention Joint Fund (Grant No. ZR2022LZL004), Wu Jieping Medical Foundation for Clinical Scientific Research (Grant No. 320.6750.2021‐14‐7) and Beijing Science and Technology Innovation Fund (Grant No. KC2021‐ZZ‐0010–1). The funding sources had no involvement in the study.

## CONFLICT OF INTEREST STATEMENT

The authors have no relevant financial or nonfinancial interests to disclose.

## PATIENT CONSENT FOR PUBLICATION

The section is not relevant to my manuscript, so it is ‘Not applicable’ for this section.

## ETHICAL STATEMENTS

This is a database‐based retrospective study, and hence informed consent and approval by institutional ethics committee are exempted.

## Supporting information


Figure S1.

Figure S2.

Table S1.
Table S2.

Data S1.

## Data Availability

The datasets analyzed during the current study are available in the SEER database using SEER*Stat software version 8.3.9 (http://www.seer.cancer.gov/seerstat) and can also be obtained by emailing the corresponding author.
