# Supplementary figures and images for: The metastasis patterns and their prognostic features in patients with de novo metastatic breast cancer of different ages
Source: Cancer Med. 2023 Sep 8;12(18):18850–60. doi: 10.1002/cam4.6509 (PMC10557883; doi:10.1002/cam4.6509)

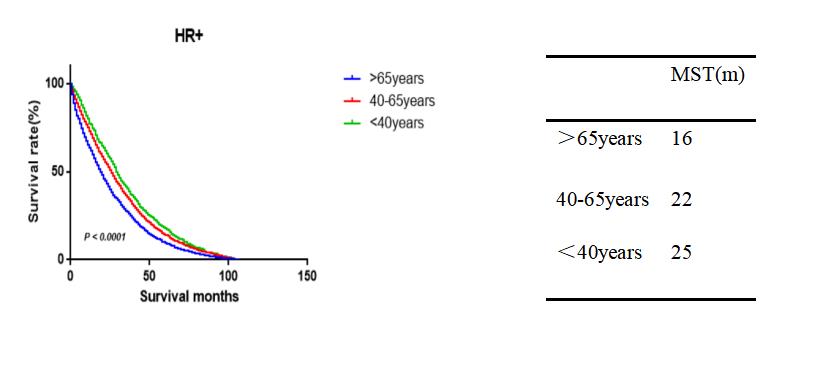

Supplement: Supplementary file 1 — Figure S1. [file CAM4-12-18850-s002.jpg]

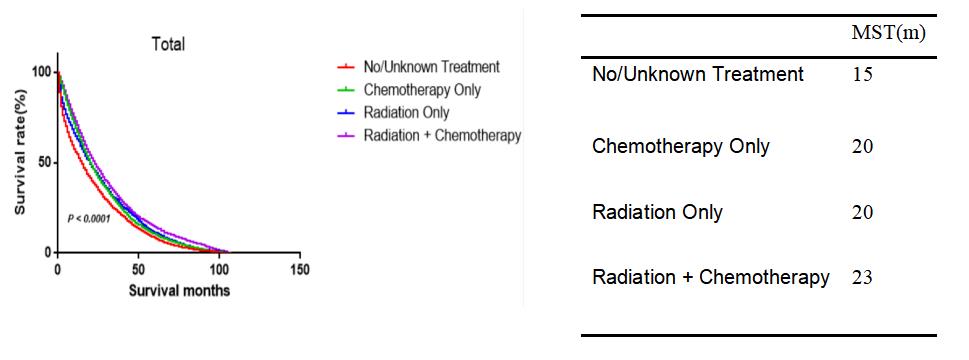

Supplement: Supplementary file 2 — Figure S2. [file CAM4-12-18850-s001.jpg]
