# Supplementary material for: The metastasis patterns and their prognostic features in patients with de novo metastatic breast cancer of different ages
Source: Cancer Med. 2023 Sep 8;12(18):18850–60. doi: 10.1002/cam4.6509 (PMC10557883; doi:10.1002/cam4.6509)
Supplement: Supplementary file 3 — Table S1. Table S2. [file CAM4-12-18850-s003.docx]

**Supplementary Table 1:** Effect of treatment for metastatic sites in de novo metastatic breast cancer patients of different ages.

|  | | | <40years | | 40-65years | | >65years | | Total | |
| --- | --- | --- | --- | --- | --- | --- | --- | --- | --- | --- |
|  |  |  | Univariate  OR(95%CI) *P*-value | Multivariate  OR(95%CI) *P*-value | Univariate  OR(95%CI) *P*-value | Multivariate  OR(95%CI) *P*-value | Univariate  OR(95%CI) *P*-value | Multivariate  OR(95%CI) *P*-value | Univariate  OR(95%CI) *P*-value | Multivariate  OR(95%CI) *P*-value |
| Bone metastases | Radiation | No/Unknown | 1(reference) | 1(reference) | 1(reference) | 1(reference) | 1(reference) | 1(reference) | 1(reference) | 1(reference) |
|  |  | Yes | **1.573 (1.281, 1.932) 0.00002** | **1.596 (1.281, 1.987) 0.00003** | **1.610 (1.485, 1.746) <0.00001** | **1.663 (1.527, 1.812) <0.00001** | **2.140 (1.933, 2.369) <0.00001** | **2.256 (2.028, 2.510) <0.00001** | **1.782 (1.678, 1.893) <0.00001** | **1.853 (1.739, 1.974) <0.00001** |
|  | Chemotherapy | No/Unknown | 1(reference) | 1(reference) | 1(reference) | 1(reference) | 1(reference) | 1(reference) | 1(reference) | 1(reference) |
|  |  | Yes | **0.467 (0.344, 0.634) <0.00001** | **0.601 (0.430, 0.840) 0.00285** | **0.609 (0.560, 0.661) <0.00001** | **0.774 (0.706, 0.847) <0.00001** | **0.812 (0.747, 0.882) <0.00001** | 0.956 (0.871, 1.048) 0.33542 | **0.692 (0.653, 0.732) <0.00001** | **0.849 (0.798, 0.905) <0.00001** |
| Lung metastases | Radiation | No/Unknown | 1(reference) | 1(reference) | 1(reference) | 1(reference) | 1(reference) | 1(reference) | 1(reference) | 1(reference) |
|  |  | Yes | **0.672 (0.531, 0.850) 0.00093** | **0.704 (0.551, 0.899) 0.00493** | **0.730 (0.672, 0.792) <0.00001** | **0.752 (0.691, 0.818) <0.00001** | **0.684 (0.621, 0.754) <0.00001** | **0.686 (0.621, 0.758) <0.00001** | **0.708 (0.666, 0.752) <0.00001** | **0.722 (0.678, 0.768) <0.00001** |
|  | Chemotherapy | No/Unknown | 1(reference) | 1(reference) | 1(reference) | 1(reference) | 1(reference) | 1(reference) | 1(reference) | 1(reference) |
|  |  | Yes | 0.878 (0.651, 1.184) 0.39360 | 0.749 (0.538, 1.041) 0.08569 | 1.024 (0.944, 1.111) 0.56494 | 0.974 (0.891, 1.064) 0.55683 | 1.017 (0.935, 1.105) 0.69960 | 0.920 (0.839, 1.008) 0.07443 | 1.015 (0.958, 1.075) 0.61013 | 0.941 (0.883, 1.001) 0.05481 |
| Live rmetastases | Radiation | No/Unknown | 1(reference) | 1(reference) | 1(reference) | 1(reference) | 1(reference) | 1(reference) | 1(reference) | 1(reference) |
|  |  | Yes | **0.492 (0.400, 0.607) <0.00001** | **0.468 (0.375, 0.583) <0.00001** | **0.587 (0.539, 0.639) <0.00001** | **0.608 (0.557, 0.664) <0.00001** | **0.625 (0.555, 0.703) <0.00001** | **0.638 (0.565, 0.721) <0.00001** | **0.588 (0.550, 0.628) <0.00001** | **0.603 (0.564, 0.645) <0.00001** |
|  | Chemotherapy | No/Unknown | 1(reference) | 1(reference) | 1(reference) | 1(reference) | 1(reference) | 1(reference) | 1(reference) | 1(reference) |
|  |  | Yes | **1.470 (1.109, 1.949) 0.00743** | 1.353 (0.994, 1.843) 0.05500 | **1.193 (1.097, 1.297) 0.00004** | 1.093 (0.998, 1.197) 0.05571 | **1.323 (1.202, 1.457) <0.00001** | **1.203 (1.082, 1.338) 0.00066** | **1.258 (1.182, 1.338) <0.00001** | **1.145 (1.070, 1.225) 0.00009** |
| Brain metastases | Radiation | No/Unknown | 1(reference) | 1(reference) | 1(reference) | 1(reference) | 1(reference) | 1(reference) | 1(reference) | 1(reference) |
|  |  | Yes | **2.811 (1.904, 4.151) <0.00001** | **3.277 (2.175, 4.938) <0.00001** | **4.446 (3.876, 5.100) <0.00001** | **5.058 (4.390, 5.827) <0.00001** | **3.930 (3.344, 4.619) <0.00001** | **4.389 (3.712, 5.189) <0.00001** | **4.116 (3.721, 4.552) <0.00001** | **4.649 (4.190, 5.159) <0.00001** |
|  | Chemotherapy | No/Unknown | 1(reference) | 1(reference) | 1(reference) | 1(reference) | 1(reference) | 1(reference) | 1(reference) | 1(reference) |
|  |  | Yes | 0.651 (0.410, 1.035) 0.06934 | 0.611 (0.363, 1.028) 0.06357 | **0.857 (0.750, 0.980) 0.02448** | **0.761 (0.658, 0.881) 0.00025** | 0.992 (0.841, 1.169) 0.92162 | 0.928 (0.775, 1.110) 0.41175 | **0.895 (0.808, 0.992) 0.03400** | **0.813 (0.728, 0.909) 0.00028** |

**Supplementary Table 2:** Effect of subtypes for metastatic sites in de novo metastatic breast cancer patients of different ages.

| .  Variable | | | HR+/HER2- | | HR-/HER2- | | HR-/HER2+ | | HR+/HER2+ | | Unknown | |
| --- | --- | --- | --- | --- | --- | --- | --- | --- | --- | --- | --- | --- |
|  |  |  | Univariate  OR(95%CI) *P*-value | Multivariate  OR(95%CI) *P*-value | Univariate  OR(95%CI) | Multivariate  OR(95%CI) *P*-value | Univariate  OR(95%CI) *P*-value | Multivariate  OR(95%CI) *P*-value | Univariate  OR(95%CI) *P*-value | Multivariate  OR(95%CI) *P*-value | Univariate  OR(95%CI) *P*-value | Multivariate  OR(95%CI) *P*-value |
| Bone metastases | Radiation | No/Unknown | 1(reference) | 1(reference) | 1(reference) | 1(reference) | 1(reference) | 1(reference) | 1(reference) | 1(reference) | 1(reference) | 1(reference) |
|  |  | Yes | **1.900 (1.733, 2.082) <0.00001** | **1.949 (1.775, 2.140) <0.00001** | **1.711 (1.465, 1.998) <0.00001** | **1.799 (1.534, 2.109) <0.00001** | **1.448 (1.197, 1.752) 0.00014** | **1.444 (1.188, 1.755) 0.00022** | **1.684 (1.447, 1.959) <0.00001** | **1.707 (1.462, 1.993) <0.00001** | **2.442 (1.990, 2.996) <0.00001** | **2.411 (1.956, 2.972) <0.00001** |
|  | Chemotherapy | No/Unknown | 1(reference) | 1(reference) | 1(reference) | 1(reference) | 1(reference) | 1(reference) | 1(reference) | 1(reference) | 1(reference) | 1(reference) |
|  |  | Yes | **0.821 (0.757, 0.890) <0.00001** | **0.799 (0.733, 0.872) <0.00001** | 0.920 (0.779, 1.086) 0.32417 | 0.963 (0.803, 1.155) 0.68751 | 0.999 (0.794, 1.256) 0.99098 | 0.932 (0.726, 1.197) 0.58289 | 0.867 (0.741, 1.015) 0.07555 | 0.846 (0.713, 1.003) 0.05387 | 1.055 (0.905, 1.230) 0.49490 | 0.954 (0.804, 1.132) 0.58722 |
| Lung metastases | Radiation | No/Unknown | 1(reference) | 1(reference) | 1(reference) | 1(reference) | 1(reference) | 1(reference) | 1(reference) | 1(reference) | 1(reference) | 1(reference) |
|  |  | Yes | **0.691 (0.635, 0.752) <0.00001** | **0.714 (0.654, 0.779) <0.00001** | **0.766 (0.653, 0.898) 0.00102** | **0.816 (0.692, 0.961) 0.01514** | **0.807 (0.660, 0.987) 0.03678** | 0.846 (0.687, 1.041) 0.11430 | **0.643 (0.551, 0.751) <0.00001** | **0.684 (0.583, 0.803) <0.00001** | **0.641 (0.530, 0.774) <0.00001** | **0.643 (0.528, 0.782) 0.00001** |
|  | Chemotherapy | No/Unknown | 1(reference) | 1(reference) | 1(reference) | 1(reference) | 1(reference) | 1(reference) | 1(reference) | 1(reference) | 1(reference) | 1(reference) |
|  |  | Yes | 0.967 (0.895, 1.045) 0.39954 | 1.050 (0.965, 1.143) 0.25813 | **0.720 (0.610, 0.851) 0.00012** | 0.851 (0.708, 1.024) 0.08757 | **0.603 (0.478, 0.760) 0.00002** | **0.668 (0.518, 0.861) 0.00185** | **0.823 (0.703, 0.964) 0.01547** | 0.945 (0.796, 1.121) 0.51443 | **0.806 (0.689, 0.942) 0.00671** | **0.795 (0.667, 0.947) 0.01008** |
| Live rmetastases | Radiation | No/Unknown | 1(reference) | 1(reference) | 1(reference) | 1(reference) | 1(reference) | 1(reference) | 1(reference) | 1(reference) | 1(reference) | 1(reference) |
|  |  | Yes | **0.699 (0.634, 0.771) <0.00001** | **0.665 (0.602, 0.735) <0.00001** | **0.673 (0.563, 0.806) 0.00002** | **0.669 (0.558, 0.803) 0.00002** | **0.533 (0.438, 0.648) <0.00001** | **0.514 (0.419, 0.630) <0.00001** | **0.528 (0.454, 0.614) <0.00001** | **0.505 (0.433, 0.589) <0.00001** | **0.578 (0.468, 0.713) <0.00001** | **0.541 (0.436, 0.672) <0.00001** |
|  | Chemotherapy | No/Unknown | 1(reference) | 1(reference) | 1(reference) | 1(reference) | 1(reference) | 1(reference) | 1(reference) | 1(reference) | 1(reference) | 1(reference) |
|  |  | Yes | **1.565 (1.431, 1.712) <0.00001** | **1.441 (1.309, 1.586) <0.00001** | 0.887 (0.739, 1.064) 0.19748 | 0.828 (0.678, 1.011) 0.06335 | **0.670 (0.532, 0.843) 0.00062** | **0.622 (0.483, 0.801) 0.00023** | 1.137 (0.974, 1.329) 0.10442 | 1.058 (0.894, 1.251) 0.51306 | 1.113 (0.946, 1.310) 0.19596 | 1.004 (0.838, 1.203) 0.96520 |
| Brain metastases | Radiation | No/Unknown | 1(reference) | 1(reference) | 1(reference) | 1(reference) | 1(reference) | 1(reference) | 1(reference) | 1(reference) | 1(reference) | 1(reference) |
|  |  | Yes | **4.177 (3.543, 4.925) <0.00001** | **4.401 (3.724, 5.203) <0.00001** | **5.349 (4.162, 6.874) <0.00001** | **5.462 (4.223, 7.065) <0.00001** | **7.868 (5.692, 10.877) <0.00001** | **9.084 (6.461, 12.772) <0.00001** | **4.576 (3.540, 5.914) <0.00001** | **4.813 (3.700, 6.261) <0.00001** | **2.863 (2.220, 3.692) <0.00001** | **2.875 (2.208, 3.743) <0.00001** |
|  | Chemotherapy | No/Unknown | 1(reference) | 1(reference) | 1(reference) | 1(reference) | 1(reference) | 1(reference) | 1(reference) | 1(reference) | 1(reference) | 1(reference) |
|  |  | Yes | 0.903 (0.773, 1.055) 0.19928 | 0.893 (0.757, 1.054) 0.18047 | **0.679 (0.528, 0.874) 0.00265** | **0.582 (0.440, 0.771) 0.00016** | 0.700 (0.500, 0.981) 0.03823 | 0.729 (0.502, 1.058) 0.09623 | **0.625 (0.485, 0.806) 0.00029** | **0.620 (0.472, 0.816) 0.00064** | 1.372 (1.067, 1.764) 0.01367 | 1.255 (0.949, 1.658) 0.11103 |
